# Roles of NAD^**+**^, PARP-1, and Sirtuins in Cell Death, Ischemic Brain Injury, and Synchrotron Radiation X-Ray-Induced Tissue Injury

**DOI:** 10.1155/2013/691251

**Published:** 2013-12-10

**Authors:** Weihai Ying

**Affiliations:** ^1^Med-X Research Institute and School of Biomedical Engineering, Shanghai Jiao Tong University, 1954 Hua Shan Road, Shanghai 200032, China; ^2^Department of Neurology, Ruijin Hospital, Shanghai Jiao Tong University School of Medicine, Shanghai 200030, China

## Abstract

NAD^+^ plays crucial roles in a variety of biological processes including energy metabolism, aging, and calcium homeostasis. Multiple studies have also shown that NAD^+^ administration can profoundly decrease oxidative cell death and ischemic brain injury. A number of recent studies have further indicated that NAD^+^ administration can decrease ischemic brain damage, traumatic brain damage and synchrotron radiation X-ray-induced tissue injury by such mechanisms as inhibiting inflammation, decreasing autophagy, and reducing DNA damage. Our latest study that applies nano-particles as a NAD^+^ carrier has also provided first direct evidence demonstrating a key role of NAD^+^ depletion in oxidative stress-induced ATP depletion. Poly(ADP-ribose) polymerase-1 (PARP-1) and sirtuins are key NAD^+^-consuming enzymes that mediate multiple biological processes. Recent studies have provided new information regarding PARP-1 and sirtuins in cell death, ischemic brain damage and synchrotron radiation X-ray-induced tissue damage. These findings have collectively supported the hypothesis that NAD^+^ metabolism, PARP-1 and sirtuins play fundamental roles in oxidative stress-induced cell death, ischemic brain injury, and radiation injury. The findings have also supported “the Central Regulatory Network Hypothesis”, which proposes that a fundamental network that consists of ATP, NAD^+^ and Ca^2+^ as its key components is the essential network regulating various biological processes.

## 1. Introduction

Increasing evidence has indicated that NAD^+^ plays important roles not only in energy metabolism and mitochondrial functions but also in aging, gene expression, calcium homeostasis, and immune functions [1–3]. Because cell death plays pivotal roles in multiple biological processes and major diseases, it is of critical importance to generalize the information regarding the roles of NAD^+^ and NAD^+^-dependent enzymes, such as PARP-1, sirtuins, and CD38, in cell death. Brain ischemia is one of the major causes of death and disability around the world [4]. A number of studies have also suggested that NAD^+^ metabolism and NAD^+^-dependent enzymes may play significant roles in ischemic brain damage [1, 2, 5]. For examples, administration of either NAD^+^ [6] or PARP inhibitors [7] has been shown to profoundly decrease ischemic brain damage.

In recent years, the information regarding the roles of NAD^+^, PARP-1, and sirtuins in various biological functions has been rapidly increasing [8–14]. A number of recent studies have also suggested novel mechanisms underlying the roles of NAD^+^, PARP-1, and sirtuins in cell death and ischemic brain damage [8, 12, 15, 16]. Based on these pieces of information, it is tempting for us to propose our hypothesis that NAD^+^, PARP-1, and sirtuins play fundamental roles in cell death, ischemic brain damage, and radiation injury. The major goal of this paper is to generalize the current findings on this topic, which appear to support our hypothesis. The information has also suggested that NAD^+^ metabolism, PARP-1 and sirtuins may become promising therapeutic targets for cerebral ischemia and radiation damage. In this overview, the knowledge gaps in this field would be identified, which would suggest valuable research directions of this increasingly significant research field.

## 2. NAD^+^ in Cell Death, Ischemic Brain Injury, and SR X-Ray-Induced Tissue Injury

### 2.1. Roles of  NAD^+^ in Cell Death

In 2003, our study provided the first evidence suggesting that NAD^+^ is a potent cytoprotective agent: NAD^+^ treatment was shown to dramatically decrease astrocyte death induced by a genotoxic agent [17]. Since then, cumulating evidence has compellingly indicated that NAD^+^ can profoundly decrease the death of multiple cell types including neurons, astrocytes, myocytes, and PC12 cells, which were induced by oxidative stress [18] or such insults as oxygen-glucose deprivation [19] and zinc [20].

Recent studies have suggested that NAD^+^ treatment can prevent not only necrosis but also apoptotic changes and autophagy. Our study has suggested that NAD^+^ treatment can significantly decrease multiple rotenone-induced apoptotic changes of PC12 cells [21]. NAD^+^ treatment was also shown to decrease staurosporine-induced caspase activation [22]. Our recent study has shown that NAD^+^ administration can markedly decrease autophagy in the brains in a mouse model of transient brain ischemia [23]. However, it remains unclear what are the mechanisms underlying the effects of NAD^+^ administration on autophagy in ischemic brains. It is also warranted to determine if NAD^+^ administration may affect the apoptotic changes in cerebral ischemia.

Our latest study has applied nanoparticles to carry NAD^+^ into both primary astrocyte cultures and differentiated PC12 cells, which has shown that the NAD^+^-carrying nanoparticles can effectively carry NAD^+^ into the cells [24]. The NAD^+^-carrying nanoparticles can not only restore the intracellular NAD^+^ and ATP levels in H_2_O_2_-treated cells but also significantly decrease H_2_O_2_-induced cell death [24]. Moreover, our experimental results have excluded the possibility that the protective effect may result from the effects of extracellular NAD^+^ released from the NAD^+^-carrying nanoparticles. This study has also provided the first direct evidence demonstrating that the oxidative stress-induced reduction of intracellular ATP is mediated by the oxidative stress-induced reduction of the intracellular NAD^+^.

The previous cell culture studies have suggested the following mechanisms underlying the protective effects of NAD^+^ on the cell death induced by oxidative stress, genotoxic agents, and zinc: first, NAD^+^ treatment can prevent genotoxic agent-induced mitochondrial permeability transition (MPT)—an important factor mediating cell death [18]. Second, NAD^+^ treatment can prevent genotoxic agent-induced inhibition of ATP depletion and glycolysis [17, 18, 25], probably due to the fact that cytosolic NAD^+^ is required for GAPDH—a key enzyme in glycolysis. Third, it has been suggested that NAD^+^ can decrease myocyte death by activating SIRT1 [26].

Interestingly, while NAD^+^ enhances the survival of normal cells under stress conditions, we have found that NAD^+^ [27], as well as NADH [28] and NADPH [29], can selectively decrease the survival of multiple types of tumor cells. The mechanisms underlying the NAD^+^-induced decrease in tumor cell survival include increased oxidative stress and opening of P2X7 receptors, because both antioxidants and P2X7 receptor antagonists can prevent the NAD^+^-induced decrease in tumor cell survival [27]. Our study has also indicated that NAD^+^ can decrease the survival of Neuro2a cells by inducing autophagy and oxidative stress [30]. Because it can both decrease tumor cell survival and protect normal cells, NAD^+^ may hold significant therapeutic potential for cancer. Our latest study has shown that NAD^+^ administration can decrease the liver injury induced by certain anticancer drugs (unpublished findings), which has further highlighted the potential of NAD^+^ for its applications in cancer treatment.

In summary, cumulating evidence has indicated that NAD^+^ could be used to decrease the death of normal cells under various conditions, which have highlighted the therapeutic potential of NAD^+^. However, while our understandings on the roles of NAD^+^ in cell death have been significantly increased, the answers of a number of major questions on this topic remain unanswered. Future studies are necessary to further investigate the mechanisms underlying the preventive effects of NAD^+^ on the various modes of cell death in both *in vitro* and *in vivo* models of cell death.

### 2.2. Roles of NAD^+^ in Brain Ischemia

Using a rat model of transient focal brain ischemia, we provided the first evidence suggesting that NAD^+^ may become a new agent for treating brain ischemia [6]: intranasal administration of NAD^+^ decreased the infarct formation of rats by approximately 85% even when NAD^+^ was administered at 2 hrs after ischemic onset [6]. Our recent study has also found that NAD^+^ administration can reduce the brain injury in a mouse model of transient focal ischemia [31], which may partially result from the capacity of NAD^+^ to inhibit autophagy [23]. A recent study has also reported that intranasal NAD^+^ administration can significantly decrease traumatic brain injury and inhibit the inflammatory responses in the traumatic brain [32]. Moreover, a study using an animal model of myocardial ischemia has also shown that specific cardiac overexpression of nicotinamide phosphoribosyltransferase—a key enzyme for NAD^+^ synthesis—can increase the NAD^+^ content in the heart, which could result in decreased myocardial infarction [33]. Collectively, increasing evidence has indicated that NAD^+^ may become a promising therapeutic agent for both cerebral ischemia and myocardial ischemia.

It has been shown that male mice had higher baseline NAD^+^ levels, compared to those of female mice [34]. Because NAD^+^ plays important roles in various biological functions, the significant differences between male and female mice in the NAD^+^ levels have implicated that the NAD^+^-dependent biological functions of male mice may be significantly different from those of female mice. This difference in the basal levels of NAD^+^ in the brain might be one of the mechanisms underlying the major differences of ischemic bran injury between male and female animals [35, 36].

Animal studies have suggested the following major mechanisms regarding the protective effects of NAD^+^ on brain ischemia and head trauma: first, NAD^+^ can produce inhibition of autophagy in a mouse model of brain ischemia [23]. Since autophagy plays a significant role in the brain injury in the animal model of brain ischemia [23], NAD^+^ administration could decrease ischemic brain injury partially by inhibiting autophagy. Second, it has been reported that NAD^+^ administration can lead to inhibition of inflammation in an animal model of head trauma [32]. Since inflammation plays an important role in traumatic brain damage [37], NAD^+^ could decrease traumatic brain damage at least partially by inhibiting inflammation. However, the mechanism underlying this effect is unclear. A latest study has suggested a possible mechanism by which NAD^+^ mediates inflammatory responses [38]. The study indicated that NAD^+^ depletion could lead to reduced deacetylation of p65 subunit of NF*κ*B by producing decreased activity of the NAD^+^-dependent enzyme SIRT1, thus leading to increased NF*κ*B activation and increased inflammatory responses in primary murine astrocytes [38].

There are a few pieces of information implicating that NAD^+^ administration might decrease ischemic brain damage also partially by decreasing DNA damage: a cell culture study has indicated that NAD^+^ treatment can promote DNA repair in neuronal cultures exposed to oxygen-glucose deprivation by preventing oxygen-glucose deprivation-induced inhibition of the essential base-excision repair enzymes AP endonuclease [19]. Our latest study regarding the effects of NAD^+^ administration on the liver injury induced by certain anticancer drugs also showed that NAD^+^ can markedly decrease double-strand DNA breaks in the liver of the drug-treated mice (unpublished observations).

It is necessary to elucidate the mechanisms by which NAD^+^ can cross cell membranes to enter cells, so as to elucidate the mechanisms underlying the protective effects of NAD^+^ on tissue injury. It has been indicated that NAD^+^ is transported across the plasma membranes of murine neurons by P2X7R [39]. We have also found that NADH, the reduced form of NAD^+^, can be transported across the plasma membranes of murine astrocytes by P2X7 receptors [40]. However, the study of Bruzzone et al. suggested that NAD^+^ can enter murine 3T3 fibroblasts through connexin 43 (Cx43) hemichannels [41]. In summary, previous studies have suggested that NAD^+^ can enter cells through either P2X7 receptors or Cx43 hemichannels. Future studies are warranted to elucidate the pathways by which NAD^+^ is transported across cell membranes in animal model studies.

**Figure 1:**
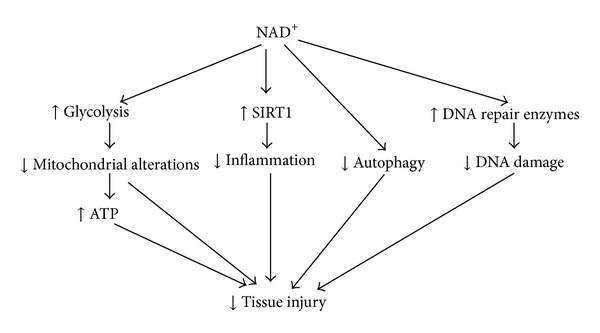
Diagrammatic presentation of the potential mechanisms underlying NAD^+^ administration-produced protective effects on the tissue injury in cerebral ischemia, head trauma, or SR X-ray irradiation.

In summary, several studies have suggested that NAD^+^ could become a neuroprotective agent not only for brain ischemia but also for such neurological diseases as head trauma. Generalizing the current information about the potential mechanisms underlying the protective effects of NAD^+^ under either *in vitro* or *in vivo* conditions, a diagram showing the potential mechanisms is presented (Figure 1). It is necessary to conduct the following three lines of work: (1) to investigate the temporal and spatial changes of the NAD^+^ metabolism in ischemic brains; (2) to further investigate the mechanisms underlying the protective effects of NAD^+^ on cerebral ischemia; and (3) to conduct preclinical studies on the effects of NAD^+^ administration on brain ischemia.

### 2.3. NAD^+^ in Synchrotron Radiation (SR) X-Ray-Induced Tissue Injury

SR X-ray has several characteristic properties: it is coherent, collimated, monochromatic, and intensely bright. These characteristic properties of SR enable the light to have rapidly increasing applications for basic biomedical research as well as medical applications [42, 43]. For examples, multiple studies have suggested that SR-based microbeam radiation therapy may become a novel approach for treating such cancers as glioma [44–46]. Although SR X-ray has great potential for its applications in medicine and biology, the fundamental mechanisms underlying SR X-ray-induced tissue injury remain unclear [47].

We have used the testes of rats as a model to test our hypothesis that NAD^+^ administration can decrease SR X-ray-induced injury of the testes [48]. We found that the SR X-ray-induced increase in double-strand DNA damage was significantly decreased by intraperitoneal administration of NAD^+^. The SR X-ray-induced increase in histological damage was also significantly decreased by the NAD^+^ administration. Collectively, our findings have indicated that SR X-ray-induced injury of the testes can be significantly attenuated by NAD^+^ administration. These results have provided a valuable basis for elucidating the mechanisms underlying SR X-ray-induced tissue injury.

### 2.4. Roles of NADH and NADPH in Cell Death

Compared with the studies regarding the roles of NADH in cell death, there has been much less information regarding the roles of NADH and NADPH in cell death. Our study has indicated that NADH can also enter astrocytes by P2X7 receptors [49] that also mediate the NAD^+^ entrance into cortical neurons [39].

We have found that treatment of C6 glioma cells can lead to decreased survival of the cells, which could be mediated by NADH treatment-induced oxidative stress and PARP activation [28]. Our study has also shown that NADPH can induce a significant decrease in the survival of C6 glioma cells, without affecting the survival of primary astrocyte cultures [29]. Our study has further indicated that NADPH decreases glioma cell survival by inducing the NADPH oxidase-dependent increase in oxidative stress and by activating PARP [29]. However, there are significant differences between the effects of NADPH and NADH on glioma cell survival: NADPH oxidase inhibitors were effective only for the effect of NADPH on the cell survival [29] but not for that of NADH [28].

There have been studies indicating that NADH could be used to treat PD patients. One study reported beneficial effects of NADH administration for approximately 80% of the patients [50], which has been substantiated by the observations from another study [51]. Potential mechanisms accounting for the effects of NADH on PD include that NADH could increase bioavailability of plasma levodopa, which is used to ameliorate the striatal dopamine deficits in PD. Moreover, NADH could enhance endogenous dopamine production, because NADH can indirectly supply reducing equivalents for dopamine synthesis [52]. It is warranted to further elucidate the mechanisms underlying the effects of NADH on PD.

## 3. Roles of PARP-1 in Cell Death, Ischemic Brain Injury, and SR X-Ray-Induced Tissue Injury

### 3.1. Roles of PARP-1 in Cell Death

PARP-1 is an abundant nuclear enzyme, which can be rapidly activated by single-strand DNA damage [53, 54]. The activated PARP-1 consumes NAD^+^ to produce poly(ADP-ribosyl)ation of target proteins such as histones and PARP-1 itself [53, 54]. PARP-1 plays important role in various biological functions including regulation of DNA repair, genomic stability, gene expression, cell cycle, and long term memory [2, 10–12, 53, 54].

Cumulative evidence has suggested that PARP-1 is a most potent NAD^+^-consuming enzyme in genotoxic agents-treated cells [2, 53]. Multiple studies have also suggested that PARP-1 plays key roles in not only programmed necrosis but also apoptosis and autophagy. Programmed necrosis is a main mode of caspase-independent programmed cell death (PCD) [55], which has been implicated in the pathology of such diseases as ischemic myocardial injury and ischemic cerebral injury [56]. The PARP-mediated programmed necrosis and the necroptosis initiated by the 55-kDa tumor necrosis factor (TNF) receptor (TNF-R1) are two most extensively studied models of programmed necrosis, which could represent distinct and independent routes of programmed necrosis [57].

Multiple studies have also suggested significant roles of PARP in apoptosis and autophagy. A recent study has suggested that PARP-1 can suppress autophagy after oxidative stress [58]. It has also been indicated that poly(ADP)ribosylation of a chromatin-bound Ca^2+^/Mg^2+^-dependent endonuclease—an enzyme involved in apoptotic DNA fragmentation—can lead to inhibition of the enzyme [59, 60]. Caspase-3 can cleave and inactivate PARP-1 during apoptosis, thus leading to decreased poly(ADP)ribosylation of Ca^2+^/Mg^2+^-dependent endonuclease and subsequent activation of the enzyme [59].

### 3.2. Roles of PARP-1 in Cerebral Ischemia

A number of studies have indicated that excessive PARP-1 activation plays a key role in ischemic brain injury of male animals. The studies using PARP-1 knockout mice have suggested that PARP-1 mediates ischemic brain damage of male mice [61, 62]; and multiple studies using various types of PARP inhibitors have also indicated a critical role of PARP-1 in the ischemic brain injury of male animals [62, 63]. Increased PARP activation has been found in the human brains after cardiac arrest [64], which implicates that PARP activation might also play a role in the ischemic brain damage in human. Multiple recent studies have further suggested that PARP-1 may become a promising therapeutic target for cerebral ischemia. One study reported that a PARP inhibitor can significantly decrease the toxic side effects of rt-PA, including the hemorrhagic transformations and reduced expression of VE-cadherin, ZO-1, and claudin-5 [65].

However, the PARP-1-based therapeutic strategy for brain ischemia has significant limitations: first, PARP-1 inhibition appears to be beneficial for relatively severe brain ischemia, while it is not beneficial for relatively mild brain ischemia [66]. Second, PARP-1 inhibition leads to decreased ischemic brain injury only in male animals, while it exacerbates ischemic brain damage in female animals [35, 36]. There are studies suggesting that androgen could be responsible for the increased PARP activation in male mice: ischemia led to a greater increase in the PARP activity in the peri-infarct region of male mice compared to female mice, and castration of male mice abolished the difference [67]. It has also been found that knockdown or inhibition of the calcium-permeable transient receptor potential M2 (TRPM2) ion channel protects male brain preferentially from ischemic brain injury [67]. This sexually dimorphic contribution of TRPM2 to ischemic brain damage may be accounted for by the sexually dimorphic contribution of PARP-1 to ischemic brain damage [67], because multiple studies have suggested that PARP-1 mediates TRPM2 opening in oxidative stress-exposed cells [68, 69].

### 3.3. Roles of PARP-1 in SR X-Ray-Induced Tissue Injury

There has been no previous report regarding the roles of PARP in SR X-ray-induced tissue injury. Our latest study tested our hypothesis that poly(ADP-ribose) polymerase (PARP) plays a significant role in SR X-ray-induced tissue damage (unpublished observations). Our study showed that SR X-ray irradiation produced dose-dependent increases in poly(ADP-ribose) (PAR) formation—an index of PARP activation, which can be prevented by the administration of the antioxidant N-acetyl cysteine (NAC), suggesting that oxidative stress mediates the SR X-ray-induced PARP activation. This finding is consistent with our previous observation suggesting that oxidative stress plays a key role in SR X-ray-induced tissue damage [70]. We further found that administration of PARP inhibitor 3-aminobenzamide decreased multiple indices of SR X-ray-induced tissue damage, including caspase-3 activation, increases in TUNEL signals, and increases in *γ*-H2AX signal—a marker of double-strand DNA breaks. The 3-aminobenzamide administration also decreased the SR X-ray-induced histological alterations of the testes. Collectively, our study has provided the first evidence suggesting that SR X-ray can induce PARP activation by generating oxidative stress, leading to various tissue injuries at least partially by inducing DNA damage and apoptotic changes.

### 3.4. Mechanisms Underlying PARP-1-Mediated Cell Death and Ischemic Brain Injury

PARP-1 inhibition could produce protective effects through several pathways: first, PARP-1 inhibition can prevent NAD^+^ depletion, thus preventing inhibition of glycolysis and such mitochondrial alterations as mitochondrial permeability transition (MPT) and mitochondrial depolarization [17, 18, 39], which could lead to restoration of ATP levels [17, 18, 39]. Second, PARP-1 inhibition could produce its protective effects by affecting Akt [71] that can produce significant cytoprotective effects by phosphorylating such apoptosis-regulatory proteins as Bad [72, 73]. Third, PARP-1 inhibition could produce protective effects by inhibiting inflammation through its effects on two critical factors in inflammation—NF*κ*B and high-mobility group protein 1 (HMGB1); PARP-1 inhibition can also lead to inhibition of NF*κ*B activity [74, 75], which produces inhibition of inflammatory responses. A latest study has suggested a mechanism underlying the effects of PARP-1 activation on inflammation: PARP-1 activation leads to decreased NAD^+^ levels and subsequent decreases in SIRT1 activity, resulting in reduced deacetylation of p65 subunit of NF*κ*B, increased NF*κ*B activation, and increased inflammatory responses in primary murine astrocytes [76]. Because PARP-1 activation plays a significant role in HMGB1 translocation [77], PARP-1 inhibition may also decrease inflammation by blocking translocation of HMGB1 out of the nucleus.

## 4. Roles of Sirtuins in Cell Death and Ischemic Brain Injury

### 4.1. Roles of Sirtuins in Cell Death

Sirtuins are the mammalian homolog of Sir2—a NAD^+^-dependent histone deacetylase that mediates the aging process of yeast [78]. In sirtuin family proteins, there are seven members, including SIRT1–SIRT7 [79]. Increasing evidence has suggested that sirtuins play fundamental roles in a variety of biological processes, including cell death, inflammation, and energy metabolism [13, 14].

#### 4.1.1. Roles of SIRT1 in Cell Death

A number of studies have suggested that SIRT1 is a critical protein for cell survival. The majority of the studies have suggested that SIRT1 activity can enhance cell survival. However, there are also studies suggesting that SIRT1 activity can exacerbate cell death. There are three mechanisms by which SIRT1 may decrease cell death: SIRT1 can produce deacetylation of p53, thus increasing degradation of p53 [80, 81], resulting in prevention of p53-mediated cell death. It has also been indicated that SIRT1 can produce a dual effect on the functions of FOXO3 [82]: SIRT1 can not only enhance the capacity of FOXO3 to induce cell cycle arrest and to produce resistance to oxidative stress but also decrease the capacity of FOXO3 to induce cell death. Moreover, SIRT1 can also prevent inflammation-induced cytotoxicity by inducing deacetylation of NF*κ*B [83, 84].

There are studies suggesting that SIRT1 activity may also exacerbate cell death under certain conditions: SIRT1 can exacerbate cell death by accelerating NAD^+^ depletion [20]. Because NF*κ*B is protective against TNF-*α*-induced cell apoptosis, SIRT1 may also exacerbate TNF-*α*-induced apoptosis by decreasing NF*κ*B activation [85].

#### 4.1.2. Roles of  SIRT2 in Cell Death

SIRT2 is a tubulin deacetylase that can produce either beneficial or detrimental effects on cell survival under various conditions. SIRT2 inhibitors were shown to reduce *α*-synuclein-induced cytotoxicity in cellular and *Drosophila* models of Parkinson's disease [86]. SIRT2 inhibition can also produce neuroprotection in models of Huntington disease, which could be mediated by a decrease in sterol biosynthesis [87]. A recent study has suggested that SIRT2 mediates programmed necrosis by modulating RIP1–RIP3 complex formation [88]. The study has also shown that the SIRT2 inhibitor AGK2 can attenuate myocardial ischemia-reperfusion injury [88]. In contrast, there are studies suggesting that SIRT2 activity is beneficial for cell survival. SIRT2 inhibition has been shown to induce apoptosis of such cell type as C6 glioma cells and HeLa cells [89, 90]. SIRT2 inhibition has also been shown to induce death of BV2 microglia [91].

Due to these seemingly contradicting effects of SIRT2 on cell survival, it appears to be critically important to further expose the mechanisms underlying the roles of SIRT2 in cell survival. The contrasting effects of SIRT2 on cell survival may be partially explained by the previous studies suggesting that SIRT2 can enhance the gene expression of both proapoptotic enzymes and antioxidation enzymes. SIRT2 activation can produce deacetylation of FOXO3a transcriptional factor, which can induce increased expression of not only the proapoptotic enzyme Bim [92] but also the antioxidation enzyme Mn-SOD [92].

Our recent studies have also suggested that the extent of SIRT2 inhibition could determine if SIRT2 inhibition is detrimental or beneficial to the survival of cells. We have found that strong inhibition of SIRT2 by 100 nM SIRT2 siRNA or 10 *μ*M AGK2, a widely used SIRT2 inhibitor [88, 93], can reduce the basal survival of PC12 cells and C6 glioma cells, thus suggesting toxic effects of strong inhibition of SIRT2 [90, 94]. However, our latest study has also suggested that mild inhibition of SIRT2 activity can significantly decrease H_2_O_2_-induced cell apoptosis (unpublished observations).

Our latest study has shown that AGK2 at 10 *μ*M—a widely used AGK2—can induce both late-stage apoptosis and necrosis of BV2 microglia, which could be mediated by PARP activation [91]. A latest study has also suggested that SIRT2 overexpression leads to inhibition of inflammation and a decrease in oxidative stress-induced death of murine macrophages, which may result from the capacity of SIRT2 to enhance the expression of the antioxidant enzymes including MnSOD, glutathione peroxidase, and catalase [95].

#### 4.1.3. Roles of SIRT3–SIRT7 in Cell Death

SIRT3, SIRT4, and SIRT5 are mitochondrial NAD^+^-dependent deacetylases [79, 96]. A number of studies have indicated the protective effects of SIRT3 on cell survival under stress conditions: SIRT3 can protect neurons from N-methyl-D-aspartate (NMDA)-induced excitotoxicity [97]. In mammalian cells treated with hypoxia or staurosporine, SIRT3 can decrease cell death by preventing mitochondrial depolarization and maintaining intracellular pH [98]. A study also suggested that SIRT3 can regulate deacetylation and turnover of 8-oxoguanine-DNA glycosylase 1—a DNA repair enzyme, thus enhancing repair of mitochondrial DNA damage leading to protection of the cells from oxidative stress-induced apoptosis [99]. Fasting can induce increased expression of nicotinamide phosphoribosyl transferase, which can prevent apoptosis by activating both SIRT3 and SIRT4 [100]. A study has also indicated that SIRT3 can prevent cardiac hypertrophy by activating antioxidant defense mechanisms [101]. There have been few studies on the roles of SIRT5 in cell survival. It has been suggested that subcellular localization of SIRT5 may determine the roles of SIRT5 in cell survival [102]; SIRT5 produces proapoptotic effect when it was localized to the mitochondria of neurons and HT-22 neuroblastoma cells. However, SIRT5 produces neuroprotective effects when it is localized to both the nucleus and cytoplasm of cerebellar granule neurons.

There have been several reports indicating significant roles of SIRT6 in the death of tumor cells. It has been reported that SIRT6 confers paclitaxel and epirubicin resistance in MCF-7 cells, which has suggested that SIRT6 is a potential marker and therapeutic target for paclitaxel- and epirubicin-resistant breast cancer [103]. One study reported that SIRT6 overexpression led to apoptosis of several cancer cell lines, but not normal cells [104]. The study has also suggested that the mono-ADP-ribosyltransferase activity, but not its deacetylase activity, mediates the effects of SIRT6 on apoptosis [104]. SIRT7 is a nucleolar protein. Several studies have suggested that SIRT7 plays a significant role in both cellular stress responses and cell survival. Knockdown of SIRT7 in human osteosarcoma U2OS cells was shown to produce apoptotic cell death [105]. SIRT7-deficient primary cardiomyocytes exhibited a significant increase in basal apoptosis, which have increased susceptibility to oxidative and genotoxic stress [106].

### 4.2. Roles of Sirtuins in Ischemic Brain Injury

Because multiple studies have suggested that SIRT1 can decrease cellular and tissue injury by such mechanisms as decreasing acetylation of p53 and NF*κ*B, it is reasonable to expect that SIRT1 may produce beneficial effects in cerebral ischemia. A latest study has shown that decreased SIRT1 activity by either pharmacological or genetic approach can lead to increased ischemic brain injury in a mouse model of permanent cerebral ischemia [107], which may be mediated by the effects of the SIRT1 inhibition/deletion on acetylation of p53 [80, 81] and NF*κ*B [83, 84]. In addition, administration of a SIRT1 activator was also shown to decrease ischemic brain damage [107]. Collectively, this study has supported the hypothesis that SIRT1 plays a beneficial role in cerebral ischemia by such mechanisms as decreasing the acetylation of p53 and the p65 subunit of NF*κ*B.

A recent study has also shown that the SIRT2 inhibitor AGK2 can attenuate myocardial ischemia-reperfusion injury [88]. Because myocardial ischemia-reperfusion injury shares multiple common pathological mechanisms with ischemic brain damage, SIRT2 inhibition might also produce neuroprotective effects in cerebral ischemia. SIRT3 has been shown to protect neurons from N-methyl-D-aspartate (NMDA)-induced excitotoxicity [97], suggesting that SIRT3 might also produce beneficial effects in cerebral ischemia, because NMDA receptor-mediated excitotoxicity plays a crucial role in ischemic brain damage [108]. It is necessary to further elucidate the roles of SIRT3 in ischemic brain injury.

## 5. Roles of CD38 in Cell Death and Ischemic Brain Injury

CD38 is a NAD^+^-dependent, multifunctional ectoenzyme. The enzyme can not only generate the second messenger, cyclic ADP-ribose (cADPR) from NAD^+^, but also transport the messengers into cells [109]. cADPR is the most potent endogenous agonist of ryanodine receptors (RyR), which plays a key role in modulating intracellular Ca^2+^ concentrations [110]. CD38 is a glycoprotein found on the surface of both immune cells and nonimmune tissues [109], which is the main NADase in the brain, heart, lung, and kidney of mice [111, 112].

Multiple studies using CD38 knockout mice have suggested that CD38/cADPR system plays important roles in neutrophils death by infection [113], autoimmune diabetes [114], and renal hemodynamics and excretory function [115]. However, there have been only quite limited studies on the roles of CD38/cADPR system in CNS. A study reported that the microglia from CD38 knockout mice has marked resistance to LPS/IFN-induced activation and activation-induced cell death [116]. It has also been suggested that CD38/cADPR system mediates glutamatergic signaling between astrocytes and neurons [117]. The glutamate released from neurons can lead to increased expression of CD38, resulting in increased cADPR and [Ca^2+^]_*i*_ in astrocytes [117].

Our study has shown that CD38 siRNA induced caspase-3-dependent apoptosis of BV2 microglia [118]. Our latest study has found that inhibition of CD38/cADPR-dependent signaling by CD38 silencing or 8-bromo-cADPR, a ryanodine receptor antagonist, produced significant decreases in the intracellular ATP levels (unpublished findings).

There have been only two studies regarding the roles of CD38 in ischemic brain damage and traumatic brain damage: CD38 knockout mice showed a decrease in ischemic brain injury [119]. However, CD38 knockout mice showed an increase in traumatic brain injury [120]. These results seem to be paradoxical, since cerebral ischemia and traumatic brain injury share multiple major pathological mechanisms [121]. We speculated that a special caution should be taken in interpreting the results using CD38 knockout mice, since the NAD^+^ level was markedly increased in multiple tissues and organs of CD38 knockout mice [112]. The markedly altered NAD^+^ levels of the CD38 knockout mice could confound the interpretations of the experimental results, considering that NAD^+^ administration can decrease both ischemia brain injury [6, 23] and traumatic brain injury [122].

## 6. Conclusions

As stated above, increasing evidence has indicated crucial roles of NAD^+^ and PARP-1 in cell survival under such pathological conditions as cerebral ischemia and SR X-ray exposures. These pieces of evidence have also suggested that NAD^+^ metabolism as well as PARP-1 may become promising therapeutic targets for multiple diseases.

In his reviews published about six years ago, Ying proposed his “Central Regulatory Network Hypothesis” that suggests that NAD/NADP, ATP, and calcium consist of a fundamental regulator network for all major biological processes [2, 123]. As reviewed in these articles, cumulating evidence has supported the hypothesis that NAD^+^ and NAD^+^-related proteins such as PARP-1 play pivotal roles in cell death and tissue injury under various pathological conditions such as cerebral ischemia and SR X-ray exposures. These pieces of information have strongly supported the “Central Regulatory Network Hypothesis.”

It is expected that future studies on the roles of NAD^+^ and NAD^+^-dependent enzymes in multiple biological processes would elucidate fundamental properties of life, which would profoundly deepen our understanding about the nature of life. The following research topics may be of particular theoretical and therapeutic significance.

First, it is necessary to further investigate the roles of NAD^+^ and NAD^+^-dependent enzymes, including PARPs, sirtuins, and CD38, in multiple major diseases such as Alzheimer's disease, cancer, and diabetes.

Second, it is necessary to further investigate the mechanisms underlying the roles of NAD^+^ and NAD^+^-dependent enzymes, particularly PARPs and sirtuins, in the pathological changes in major diseases.

Third, based on previous studies regarding the roles of NAD^+^ and PARP-1 in such diseases as brain ischemia, it is warranted to initiate preclinical trials to determine the effectiveness of NAD^+^ and PARP inhibitors for treating such diseases as brain ischemia.

Fourth, it is necessary to investigate the mechanisms of NAD^+^ metabolism and NAD^+^ transport in different tissues *in vivo*.

Fifth, as proposed in the “Central Regulatory Network Hypothesis,” the interactions among the three major components of the “Central Regulatory Network,” that is, NAD^+^, ATP, and Ca^2+^, play crucial roles in regulating the various biological functions. However, so far the information on this topic is still deficient. Therefore, it is necessary to conduct the research on this topic.

It is apparent that, while we have made dramatic progresses on the understanding regarding the roles of NAD^+^ and NAD^+^-dependent enzymes in biological functions, numerous major questions on this topic remain unanswered. It can be expected that future studies on the roles of NAD^+^ and NAD^+^-dependent enzymes in biological processes would provide critical information for understanding the nature of life, which may also provide essential information for designing novel therapeutic strategies for major diseases.
